# (2*E*,4*E*,6*E*)-3-Methyl-7-(pyren-1-yl)octa-2,4,6-trienoic acid

**DOI:** 10.1107/S1600536809038409

**Published:** 2009-09-30

**Authors:** Stavros E. Bariamis, George E. Magoulas, Constantinos M. Athanassopoulos, Dionissios Papaioannou, Manolis J. Manos, Vassilios Nastopoulos

**Affiliations:** a Department of Chemistry, University of Patras, 265 04 Patras, Greece; b Department of Chemistry, University of Cyprus, 1678 Nicosia, Cyprus

## Abstract

The title compound, C_25_H_20_O_2_, was synthesized by a Wittig reaction between triphen­yl[1-(pyren-1-yl)eth­yl]phospho­nium bromide and ethyl (2*E*,4*E*)-3-methyl-6-oxohexa-2,4-dienoate, in the presence of *n*-butyl lithium, followed by saponification. It was obtained pure in the all-*trans* configuration following crystallization from ethyl acetate. The asymmetric unit contains two independent mol­ecules (*A* and *B*), which are arranged almost parallel to each other within the crystal structure. The triene chain is not coplanar with the pyrene ring system, forming dihedral angles of 52.8 (1) and 42.2 (1)° for mol­ecules *A* and *B*, respectively. Inter­molecular hydrogen bonds between the carboxyl groups of the mol­ecules link them into centrosymmetric pairs, *AA* and *BB*, each with the *R*_2_^2^(8) graph-set motif.

## Related literature

For general background on retinoids, see: Meyer *et al.* (1978 ▶); Sporn *et al.* (1994 ▶); Tian *et al.* (1997 ▶); Chaudhuri *et al.* (1999 ▶); Malpezzi *et al.* (2005 ▶). For graph-set notation, see: Bernstein *et al.* (1995 ▶).
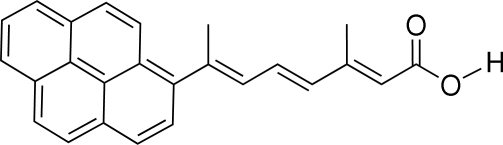


## Experimental

### 

#### Crystal data

C_25_H_20_O_2_
*M* *_r_* = 352.41
Triclinic, 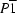

*a* = 7.5751 (7) Å
*b* = 8.5466 (7) Å
*c* = 28.458 (3) Å
α = 97.086 (7)°
β = 93.003 (8)°
γ = 97.574 (7)°
*V* = 1808.0 (3) Å^3^
*Z* = 4
Mo *K*α radiation
μ = 0.08 mm^−1^
*T* = 100 K
0.21 × 0.17 × 0.14 mm

#### Data collection

Oxford Diffraction Xcalibur-3 with Sapphire CCD diffractometer
Absorption correction: none
22799 measured reflections
6284 independent reflections
3448 reflections with *I* > 2σ(*I*)
*R*_int_ = 0.109

#### Refinement

*R*[*F* ^2^ > 2σ(*F* ^2^)] = 0.066
*wR*(*F* ^2^) = 0.160
*S* = 1.00
6284 reflections
497 parameters
2 restraints
H atoms treated by a mixture of independent and constrained refinement
Δρ_max_ = 0.33 e Å^−3^
Δρ_min_ = −0.35 e Å^−3^

### 

Data collection: *CrysAlis CCD* (Oxford Diffraction, 2008 ▶); cell refinement: *CrysAlis RED* (Oxford Diffraction, 2008 ▶); data reduction: *CrysAlis RED*; program(s) used to solve structure: *SIR92* (Altomare *et al.*, 1994 ▶); program(s) used to refine structure: *SHELXL97* (Sheldrick, 2008 ▶); molecular graphics: *PLATON* (Spek, 2009 ▶) and *Mercury* (Macrae *et al.*, 2006 ▶); software used to prepare material for publication: *WinGX* (Farrugia, 1999 ▶) and *publCIF* (Westrip, 2009 ▶).

## Supplementary Material

Crystal structure: contains datablocks I, global. DOI: 10.1107/S1600536809038409/fj2242sup1.cif

Structure factors: contains datablocks I. DOI: 10.1107/S1600536809038409/fj2242Isup2.hkl

Additional supplementary materials:  crystallographic information; 3D view; checkCIF report

## Figures and Tables

**Table 1:** Hydrogen-bond geometry (Å, °)

*D*—H⋯*A*	*D*—H	H⋯*A*	*D*⋯*A*	*D*—H⋯*A*
O2*A*—H2*A*1⋯O1*A*^i^	0.876 (18)	1.756 (19)	2.629 (3)	174 (4)
O2*B*—H2*B*1⋯O1*B*^ii^	0.858 (18)	1.79 (2)	2.624 (3)	162 (4)

Symmetry codes: (i) 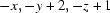; (ii) 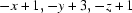.

**Fig. 1.**
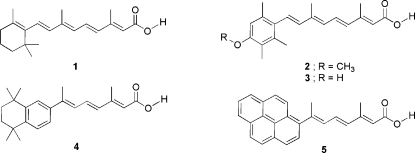
Synthetic scheme, part 1.

**Fig. 2.**
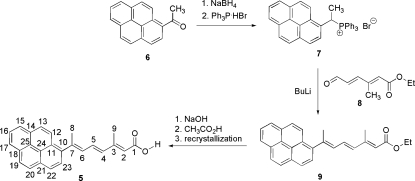
Synthetic scheme, part 2.

**Fig. 3.**
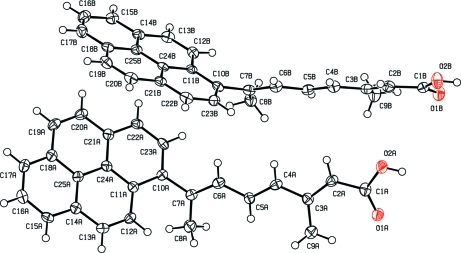
Structure of molecules A and B present in the title compound (5) with the atom-labelling scheme. Displacement ellipsoids are drawn at the 50% probability level.

**Fig. 4.**
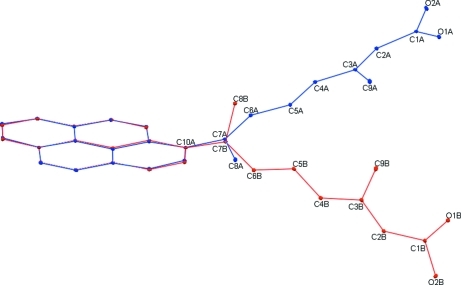
A least-squares fit of molecules A (blue) and B (red) within the asymmetric unit. The fitting fragment (the pyrene ring system) of the two molecules has an r.m.s. deviation of 0.035. Hydrogen atoms have been omitted for clarity.

## supplementary crystallographic information

### Comment

Retinoids are compounds structurally related to vitamin A which play an important role in a variety of biological functions including vision, development, reproduction and cell differentiation and have been applied successfully to the management of severe skin disorders (Sporn *et al.*, 1994; Meyer *et al.*, 1978, and references therein). They exert their effects by binding to the nuclear receptors RAR and RXR, for which *all-trans* retinoic acid (ATRA, 1) (Fig. 1) and its 9-*cis* isomer have been identified as the principal natural ligands. A huge array of analogs of ATRA have been synthesized in order to improve the therapeutic efficacy to toxicity index and to provide better selectivities for various therapeutic applications. These analogs usually involve changes in the lipophilic part of the molecules and/or the tetraene chain. A well known such example is acitretin (2) which is currently the drug of choice for treating psoriasis and has been shown to exert its effect indirectly, that is not by binding to the retinoid receptors but by displacing ATRA from its cellular binding proteins (CRABPs) (Tian *et al.*, 1997). Recently, the crystal structures of three polymorphic forms (I, II and III) of acitretin have been determined (Malpezzi *et al.*, 2005) as well the crystal structures of the acitretin analog 3 (*O*-demethylated acitretin) and ATRA analog 4, in which the double bond adjacent to the ring is restricted within an aromatic ring, in complex with CRABP II (Chaudhuri *et al.*, 1999). Both in the crystal structures of acitretin and its analog 3 the aromatic ring and the polyene chain are not coplanar but form dihedral angles of maximum 38.4° (forms I and II) and 60.8° (form III) and 56°, respectively, whereas in the crystal structure of ATRA analog 4, a charge/π-cloud interaction between the aromatic ring and Arg59 residue is identified (Chaudhuri *et al.*, 1999) which might account for the somewhat stronger binding of 4 to CRABP II binding domain. We therefore considered of interest to combine structural features from the lipophilic parts of acitretin and ATRA analog 4. Accordingly, we synthesized acitretin analog 5 bearing a pyrene ring-system in the lipophilic part of the molecule by using as key-step the Wittig reaction of triphenyl[1-(pyren-1-yl)ethyl]phosphonium bromide, readily obtained from the commercially available 1-acetylpyrene, and ethyl (2E,4E)-3-methyl-6-oxohexa-2,4-dienoate whose synthesis has been described in the literature (Meyer *et al.*, 1978).

Indeed, reduction of the commercially available 1-acetylpyrene (6) with NaBH_4_, followed by treatment of the thus obtained alcohol with Ph_3_P.HBr, provided the phosphonium salt (7) (Fig. 2). This salt was subjected to a Wittig reaction with the unsaturated aldehyde 8 (Meyer *et al.*, 1978) using *n*-BuLi as the base to obtain ester 9 as a mixture of geometric isomers. Finally, saponification and recrystallization of the thus obtained acid from ethyl acetate provided the title compound (5). Only the *all-E* isomers of compounds 8 and 9 are drawn in Figure 2.

We now wish to report the results of the X-ray crystallographic analysis of acitretin analog 5. Its asymmetric unit contains two symmetry-independent molecules (labelled A and B) which are arranged almost parallel to each other within the crystal structure and have their carboxylic ends pointing in the same direction (Fig. 3). Molecules A and B have an enantiomeric-type relationship and a least-squares fit of A (blue) and B (red) within the asymmetric unit is presented in Fig. 4. The pyrene ring system of the two independent molecules shows a planar arrangement; the r.m.s. deviation of the sixteen atoms consisting this system is 0.042 Å and 0.019 Å for A and B, respectively. The triene chain of each molecule A and B forms with the corresponding pyrene system a dihedral angle of 52.8 (1)° and 42.2 (1)°, respectively.

The carboxylic moiety of each molecule forms strong intermolecular hydrogen bonds with a neighbouring centrosymmetric molecule (Table 1), thereby linking them into elongated AA and BB dimers located on crystallographic inversion centres; those interactions can be described by the classic graph-set motif of *R*_2_^2^(8) (Bernstein *et al.*, 1995). Similar centrosymmetric hydrogen-bonded dimers have also been observed in form II of acitretin (Malpezzi *et al.*, 2005). The formation of such dimers excludes the possibility of the presence of specific supramolecular arrangements such as chains or layers. The packing of the dimers within the crystal structure is accomplished through normal van der Waals contacts.

### Experimental

To an ice-cold solution of 1-acetylpyrene (0.73 g, 3 mmol) in MeOH/diglyme (3:7, 6 ml), NaBH_4_ (0.29 g, 7.6 mmol) was added portionwise in 15 min. The resulting reaction mixture was stirred at this temperature for 45 min. Excess NaBH_4_ was destroyed by adding icechips. The product was extracted with EtOAc, the organic layer was washed twice with H_2_O, dried over Na_2_SO_4_ and evaporated to dryness to leave the corresponding alcohol (0.71 g, 96% yield) as a pale yellow solid and had *R*_f_ (PhMe) 0.11. This alcohol was treated with Ph_3_P.HBr (2.0 g, 5.76 mmol) in MeCN/THF (7:3, 6 ml) at 80 *^o^*C for 12 h. After evaporation of the solvents, trituration with Et_2_O and overnight refrigeration, the corresponding phosphonium salt 7 was obtained as pale yellow solid (1.35 g, 82%) and used as such without further purification into the following experiment. A solution of phosphonium salt 7 (1.03 g, 1.8 mmol) in THF (1.5 ml) and DMPU (0.5 ml) was cooled at -10 *^o^*C and a 1.6 *M* solution of *n*-BuLi in hexanes (1.35 ml) was added dropwise. The resulting dark red solution was left to vigorously stirring over 30 min at this temperature. Then, temperature was set at -78 *^o^*C and aldehyde 8 (0.15 g, 0.9 mmol) was added. The resulting reaction mixture was left to stir at -78 *^o^*C over 30 min and then to attain room temperature for 12 h. Excess *n*-BuLi was destroyed by careful addition of a 5% aqueous solution of NH_4_Cl to pH 7–8. The mixture was extracted with EtOAc, washed twice with H_2_O, dried over Na_2_SO_4 _and evaporated to dryness. The corresponding unsaturated ester 9 (0.09 g, 25%) was obtained in oily form after f.c.c. purification using PhMe:Hex 7:3 as the eluent (*R*_f_ 0.28) as an inseparable mixture of geometric isomers (*all-E*-9*ca* 75% of the mixture). The thus obtained ester 9 (0.09 g, 0.22 mmol) was suspended in MeOH/DMSO (6:1, 0.7 ml) and saponified with an 8 N aqueous solution of NaOH (0.11 ml) at 70 *^o^*C for 3 h. After evaporation of MeOH, the oily residue was diluted with H_2_O and acidified with glacial acetic acid to pH 5. The product was extracted with EtOAc. The organic layers were combined and washed once with a saturated aqueous solution of NaCl and twice with H_2_O, dried over Na_2_SO_4_ and evaporated to dryness to obtain the corresponding acid 5 (0.04 g, 85%) from which *all-E*-5 was obtained in 40% yield following crystallization from EtOAc. Recrystallization from EtOAc gave finally yellow crystals of *all-trans*-5 suitable for X-ray analysis; m.p. 517–518 K.

### Refinement

The H atoms of the carboxylic acid groups were located in difference Fourier maps and their positions were refined with soft distance restraints along with *U*_iso_(H) equal to 1.5*U*_eq _of their parent atoms. The methine and aromatic H atoms were placed in geometrically idealized positions [C—H = 0.93 Å and *U*_iso_(H) = 1.2*U*_eq_(C)]; the remaining methyl H atoms were constrained to an ideal geometry [C—H = 0.96 Å and *U*_iso_(H) = 1.5*U*_eq_(C)], but were allowed to rotate freely about the C—C bonds. Two low-angle reflections were omitted from the final cycles of refinement because their observed intensities were significantly lower than the calculated values, being apparently obscured by the beam stop.

### Crystal data

**Table:**

C_25_H_20_O_2_	*Z* = 4
*M**_r_* = 352.41	*F*(000) = 744
Triclinic, *P*1	*D*_x_ = 1.295 Mg m^−^^3^
Hall symbol: -P 1	Mo *K*α radiation, λ = 0.71073 Å
*a* = 7.5751 (7) Å	Cell parameters from 4369 reflections
*b* = 8.5466 (7) Å	θ = 3.0–30.3°
*c* = 28.458 (3) Å	µ = 0.08 mm^−^^1^
α = 97.086 (7)°	*T* = 100 K
β = 93.003 (8)°	Prism, colourless
γ = 97.574 (7)°	0.21 × 0.17 × 0.14 mm
*V* = 1808.0 (3) Å^3^	

### Data collection

**Table:**

Oxford Diffraction Xcalibur-3 with Sapphire CCD diffractometer	3448 reflections with *I* > 2σ(*I*)
Radiation source: Enhance (Mo) X-ray source	*R*_int_ = 0.109
graphite	θ_max_ = 25.0°, θ_min_ = 3.0°
Detector resolution: 16.0288 pixels mm^-1^	*h* = −9→9
ω and φ scans	*k* = −9→10
22799 measured reflections	*l* = −33→33
6284 independent reflections	

### Refinement

**Table:**

Refinement on *F*^2^	Primary atom site location: structure-invariant direct methods
Least-squares matrix: full	Secondary atom site location: difference Fourier map
*R*[*F*^2^ > 2σ(*F*^2^)] = 0.066	Hydrogen site location: inferred from neighbouring sites
*wR*(*F*^2^) = 0.160	H atoms treated by a mixture of independent and constrained refinement
*S* = 1.00	*w* = 1/[σ^2^(*F*_o_^2^) + (0.0717*P*)^2^] where *P* = (*F*_o_^2^ + 2*F*_c_^2^)/3
6284 reflections	(Δ/σ)_max_ < 0.001
497 parameters	Δρ_max_ = 0.33 e Å^−^^3^
2 restraints	Δρ_min_ = −0.35 e Å^−^^3^

### Special details

**Table:**

Experimental. IR (KBr, cm^-1^): 3200–2610, 2937, 2849, 1674; HPLC (40% MeCN/H_2_O to 100% MeCN, C_18_, 3.5 µm, 150x4.6 mm): t_R_ = 21.073 min; ^1^H–NMR (400 MHz, d_6_-DMSO): δ 12.12 (br. s, 1H), 8.30 (d, J = 7.2 Hz, 1H), 8.28 (d, J = 7.2 Hz, 2H), 8.18 (m, 4H) , 8.08 (t, *J* = 7.2 Hz, 1H), 7.95 (d, *J* = 8 Hz, 1H), 7.23 (dd, *J* = 11.2 and 15.2 Hz, 1H), 6.53 (d, *J* = 15.2 Hz, 1H), 6.39 (d, *J* = 11.2 Hz, 1H), 5.83 (s, 1H), 2.47 (s, 3H), 2.39 (s, 3H) p.p.m.; ESI-MS (30 eV): *m/z* 704.21 (2*M*), 353.37 (MH), 335.36 (MH—H_2_O).
Geometry. All e.s.d.'s (except the e.s.d. in the dihedral angle between two l.s. planes) are estimated using the full covariance matrix. The cell e.s.d.'s are taken into account individually in the estimation of e.s.d.'s in distances, angles and torsion angles; correlations between e.s.d.'s in cell parameters are only used when they are defined by crystal symmetry. An approximate (isotropic) treatment of cell e.s.d.'s is used for estimating e.s.d.'s involving l.s. planes.
Refinement. Refinement of *F*^2^ against ALL reflections. The weighted *R*-factor *wR* and goodness of fit *S* are based on *F*^2^, conventional *R*-factors *R* are based on *F*, with *F* set to zero for negative *F*^2^. The threshold expression of *F*^2^ > σ(*F*^2^) is used only for calculating *R*-factors(gt) *etc*. and is not relevant to the choice of reflections for refinement. *R*-factors based on *F*^2^ are statistically about twice as large as those based on *F*, and *R*- factors based on ALL data will be even larger.

### Fractional atomic coordinates and isotropic or equivalent isotropic displacement parameters (Å^2^)

**Table:**

	*x*	*y*	*z*	*U*_iso_*/*U*_eq_	
C1A	0.0508 (4)	0.8362 (4)	0.45141 (12)	0.0247 (8)	
C1B	0.4808 (4)	1.3280 (4)	0.44843 (11)	0.0243 (8)	
C2A	0.0917 (4)	0.7231 (4)	0.41240 (11)	0.0247 (8)	
H2A	0.1673	0.7649	0.3909	0.030*	
C2B	0.4635 (4)	1.2089 (4)	0.40651 (11)	0.0259 (8)	
H2B	0.4067	1.2354	0.3794	0.031*	
C3A	0.0344 (4)	0.5659 (4)	0.40331 (11)	0.0225 (8)	
C3B	0.5198 (4)	1.0650 (4)	0.40221 (11)	0.0241 (8)	
C4A	0.0898 (4)	0.4799 (4)	0.36041 (11)	0.0233 (8)	
H4A	0.1564	0.5400	0.3407	0.028*	
C4B	0.4895 (4)	0.9708 (4)	0.35600 (11)	0.0249 (8)	
H4B	0.4385	1.0179	0.3319	0.030*	
C5A	0.0542 (4)	0.3227 (4)	0.34658 (11)	0.0225 (8)	
H5A	−0.0107	0.2600	0.3660	0.027*	
C5B	0.5275 (4)	0.8214 (4)	0.34422 (11)	0.0239 (8)	
H5B	0.5766	0.7701	0.3677	0.029*	
C6A	0.1115 (4)	0.2460 (4)	0.30317 (11)	0.0243 (8)	
H6A	0.1718	0.3113	0.2836	0.029*	
C6B	0.4949 (4)	0.7392 (4)	0.29682 (11)	0.0254 (8)	
H6B	0.4503	0.7952	0.2739	0.030*	
C7A	0.0858 (4)	0.0887 (4)	0.28832 (11)	0.0210 (7)	
C7B	0.5226 (4)	0.5888 (4)	0.28218 (11)	0.0231 (8)	
C8A	−0.0034 (5)	−0.0307 (4)	0.31727 (11)	0.0316 (9)	
H8A1	0.0530	−0.0133	0.3488	0.047*	
H8A2	0.0071	−0.1363	0.3028	0.047*	
H8A3	−0.1274	−0.0186	0.3186	0.047*	
C8B	0.5888 (4)	0.4862 (4)	0.31719 (11)	0.0275 (8)	
H8B1	0.7114	0.5246	0.3273	0.041*	
H8B2	0.5788	0.3781	0.3023	0.041*	
H8B3	0.5180	0.4909	0.3442	0.041*	
C9A	−0.0803 (5)	0.4746 (4)	0.43400 (12)	0.0331 (9)	
H9A1	−0.0110	0.4082	0.4500	0.050*	
H9A2	−0.1771	0.4092	0.4149	0.050*	
H9A3	−0.1272	0.5471	0.4570	0.050*	
C9B	0.6137 (5)	0.9991 (4)	0.44129 (12)	0.0375 (10)	
H9B1	0.6380	1.0790	0.4685	0.056*	
H9B2	0.7240	0.9680	0.4309	0.056*	
H9B3	0.5394	0.9081	0.4496	0.056*	
C10A	0.1586 (4)	0.0308 (4)	0.24283 (10)	0.0202 (7)	
C10B	0.4752 (4)	0.5222 (4)	0.23171 (11)	0.0209 (7)	
C11A	0.0509 (4)	−0.0710 (4)	0.20581 (11)	0.0195 (7)	
C11B	0.5854 (4)	0.4329 (4)	0.20344 (11)	0.0190 (7)	
C12A	−0.1353 (4)	−0.1213 (4)	0.20822 (11)	0.0216 (8)	
H12A	−0.1895	−0.0907	0.2358	0.026*	
C12B	0.7587 (4)	0.4024 (4)	0.22032 (11)	0.0230 (8)	
H12B	0.8021	0.4440	0.2511	0.028*	
C13A	−0.2351 (4)	−0.2122 (4)	0.17156 (11)	0.0230 (8)	
H13A	−0.3552	−0.2448	0.1750	0.028*	
C13B	0.8592 (4)	0.3159 (4)	0.19311 (11)	0.0243 (8)	
H13B	0.9677	0.2951	0.2061	0.029*	
C14A	−0.1619 (4)	−0.2593 (4)	0.12809 (11)	0.0214 (8)	
C14B	0.8048 (4)	0.2547 (4)	0.14485 (11)	0.0228 (8)	
C15A	−0.2625 (4)	−0.3479 (4)	0.08916 (11)	0.0235 (8)	
H15A	−0.3826	−0.3830	0.0918	0.028*	
C15B	0.9078 (4)	0.1650 (4)	0.11602 (11)	0.0258 (8)	
H15B	1.0170	0.1434	0.1282	0.031*	
C16A	−0.1880 (4)	−0.3848 (4)	0.04670 (11)	0.0276 (8)	
H16A	−0.2586	−0.4422	0.0209	0.033*	
C16B	0.8500 (4)	0.1080 (4)	0.06972 (12)	0.0300 (9)	
H16B	0.9202	0.0480	0.0510	0.036*	
C17A	−0.0082 (4)	−0.3366 (4)	0.04229 (11)	0.0268 (8)	
H17A	0.0408	−0.3618	0.0135	0.032*	
C17B	0.6881 (5)	0.1392 (4)	0.05074 (12)	0.0293 (8)	
H17B	0.6503	0.0989	0.0195	0.035*	
C18A	0.0999 (4)	−0.2508 (4)	0.08053 (11)	0.0220 (8)	
C18B	0.5813 (4)	0.2301 (4)	0.07801 (11)	0.0232 (8)	
C19A	0.2858 (4)	−0.2031 (4)	0.07779 (11)	0.0255 (8)	
H19A	0.3387	−0.2341	0.0500	0.031*	
C19B	0.4127 (4)	0.2657 (4)	0.05942 (12)	0.0270 (8)	
H19B	0.3738	0.2283	0.0280	0.032*	
C20A	0.3873 (4)	−0.1144 (4)	0.11424 (11)	0.0254 (8)	
H20A	0.5081	−0.0840	0.1110	0.030*	
C20B	0.3109 (4)	0.3511 (4)	0.08627 (11)	0.0246 (8)	
H20B	0.2038	0.3735	0.0730	0.030*	
C21A	0.3126 (4)	−0.0664 (4)	0.15760 (11)	0.0209 (7)	
C21B	0.3634 (4)	0.4090 (4)	0.13516 (11)	0.0218 (8)	
C22A	0.4134 (4)	0.0312 (4)	0.19507 (11)	0.0222 (7)	
H22A	0.5341	0.0638	0.1924	0.027*	
C22B	0.2594 (4)	0.4966 (4)	0.16391 (11)	0.0238 (8)	
H22B	0.1513	0.5194	0.1513	0.029*	
C23A	0.3358 (4)	0.0796 (4)	0.23598 (11)	0.0214 (8)	
H23A	0.4052	0.1477	0.2600	0.026*	
C23B	0.3125 (4)	0.5504 (4)	0.21052 (11)	0.0230 (8)	
H23B	0.2384	0.6075	0.2288	0.028*	
C24A	0.1296 (4)	−0.1163 (4)	0.16294 (11)	0.0194 (7)	
C24B	0.5297 (4)	0.3771 (4)	0.15525 (11)	0.0189 (7)	
C25A	0.0224 (4)	−0.2094 (4)	0.12396 (11)	0.0195 (7)	
C25B	0.6370 (4)	0.2868 (4)	0.12604 (11)	0.0210 (7)	
O1A	−0.0314 (3)	0.8043 (3)	0.48587 (8)	0.0293 (6)	
O1B	0.5355 (3)	1.3133 (3)	0.48846 (8)	0.0316 (6)	
O2A	0.1129 (3)	0.9852 (3)	0.44623 (8)	0.0320 (6)	
H2A1	0.092 (5)	1.054 (4)	0.4701 (10)	0.048*	
O2B	0.4270 (3)	1.4636 (3)	0.43819 (8)	0.0342 (6)	
H2B1	0.443 (5)	1.520 (4)	0.4655 (8)	0.051*	

### Atomic displacement parameters (Å^2^)

**Table:**

	*U*^11^	*U*^22^	*U*^33^	*U*^12^	*U*^13^	*U*^23^
C1A	0.032 (2)	0.019 (2)	0.0256 (19)	0.0097 (16)	0.0007 (15)	0.0053 (16)
C1B	0.0243 (18)	0.026 (2)	0.0222 (19)	0.0010 (16)	0.0056 (14)	0.0036 (16)
C2A	0.0261 (18)	0.024 (2)	0.0265 (19)	0.0080 (16)	0.0072 (14)	0.0059 (16)
C2B	0.0264 (19)	0.028 (2)	0.0233 (18)	0.0018 (16)	0.0009 (14)	0.0070 (16)
C3A	0.0234 (18)	0.019 (2)	0.0263 (19)	0.0057 (15)	0.0008 (14)	0.0034 (16)
C3B	0.0202 (18)	0.028 (2)	0.0245 (18)	−0.0003 (15)	0.0030 (14)	0.0066 (16)
C4A	0.0213 (18)	0.026 (2)	0.0245 (18)	0.0070 (15)	0.0061 (13)	0.0064 (16)
C4B	0.0233 (18)	0.028 (2)	0.0246 (18)	0.0052 (16)	0.0005 (14)	0.0068 (16)
C5A	0.0236 (18)	0.020 (2)	0.0232 (18)	0.0024 (15)	0.0013 (13)	0.0013 (15)
C5B	0.0217 (18)	0.028 (2)	0.0235 (18)	0.0035 (16)	0.0037 (13)	0.0086 (16)
C6A	0.0230 (18)	0.029 (2)	0.0230 (18)	0.0053 (16)	0.0030 (14)	0.0065 (16)
C6B	0.0232 (18)	0.027 (2)	0.0270 (19)	0.0063 (16)	0.0012 (14)	0.0061 (16)
C7A	0.0216 (18)	0.020 (2)	0.0232 (18)	0.0058 (15)	0.0032 (13)	0.0058 (15)
C7B	0.0204 (18)	0.022 (2)	0.0284 (19)	0.0025 (15)	0.0036 (14)	0.0071 (16)
C8A	0.039 (2)	0.031 (2)	0.0238 (19)	0.0009 (18)	0.0032 (15)	0.0039 (17)
C8B	0.034 (2)	0.022 (2)	0.0266 (19)	0.0045 (16)	0.0013 (15)	0.0047 (16)
C9A	0.038 (2)	0.034 (2)	0.0271 (19)	0.0027 (18)	0.0068 (16)	0.0025 (17)
C9B	0.047 (2)	0.035 (2)	0.031 (2)	0.0151 (19)	−0.0033 (17)	−0.0005 (18)
C10A	0.0211 (18)	0.0209 (19)	0.0197 (17)	0.0050 (15)	0.0022 (13)	0.0047 (15)
C10B	0.0226 (18)	0.0126 (18)	0.0274 (19)	0.0003 (14)	0.0008 (14)	0.0054 (15)
C11A	0.0235 (18)	0.0146 (18)	0.0222 (18)	0.0061 (15)	0.0023 (13)	0.0054 (14)
C11B	0.0196 (17)	0.0115 (18)	0.0261 (18)	−0.0002 (14)	0.0041 (13)	0.0054 (14)
C12A	0.0225 (18)	0.023 (2)	0.0219 (18)	0.0086 (15)	0.0061 (14)	0.0055 (15)
C12B	0.0250 (18)	0.020 (2)	0.0225 (18)	0.0004 (15)	−0.0018 (14)	0.0031 (15)
C13A	0.0198 (17)	0.024 (2)	0.0272 (19)	0.0036 (15)	0.0039 (14)	0.0082 (16)
C13B	0.0185 (18)	0.024 (2)	0.033 (2)	0.0057 (15)	−0.0005 (14)	0.0095 (16)
C14A	0.0252 (19)	0.0176 (19)	0.0230 (18)	0.0067 (15)	0.0013 (14)	0.0051 (15)
C14B	0.0243 (18)	0.0164 (19)	0.0290 (19)	0.0022 (15)	0.0042 (14)	0.0078 (15)
C15A	0.0220 (18)	0.0181 (19)	0.0304 (19)	−0.0004 (15)	0.0012 (14)	0.0065 (15)
C15B	0.0265 (19)	0.022 (2)	0.031 (2)	0.0061 (16)	0.0046 (15)	0.0095 (16)
C16A	0.031 (2)	0.023 (2)	0.027 (2)	0.0039 (16)	−0.0041 (15)	0.0002 (16)
C16B	0.036 (2)	0.023 (2)	0.034 (2)	0.0096 (17)	0.0126 (16)	0.0063 (17)
C17A	0.035 (2)	0.024 (2)	0.0223 (19)	0.0089 (17)	0.0054 (15)	0.0011 (15)
C17B	0.041 (2)	0.020 (2)	0.0261 (19)	0.0025 (17)	0.0035 (16)	0.0016 (16)
C18A	0.0287 (19)	0.0161 (19)	0.0232 (18)	0.0075 (15)	0.0033 (14)	0.0055 (15)
C18B	0.0280 (19)	0.0159 (19)	0.0265 (19)	−0.0014 (15)	0.0048 (14)	0.0094 (15)
C19A	0.0272 (19)	0.027 (2)	0.0260 (19)	0.0119 (16)	0.0093 (14)	0.0081 (16)
C19B	0.030 (2)	0.023 (2)	0.0264 (19)	−0.0019 (16)	−0.0045 (15)	0.0058 (16)
C20A	0.0243 (18)	0.024 (2)	0.031 (2)	0.0096 (16)	0.0095 (15)	0.0082 (16)
C20B	0.0233 (18)	0.024 (2)	0.0276 (19)	0.0026 (16)	−0.0025 (14)	0.0098 (16)
C21A	0.0213 (18)	0.0156 (18)	0.0270 (18)	0.0066 (15)	0.0024 (14)	0.0039 (15)
C21B	0.0199 (18)	0.0172 (19)	0.0290 (19)	0.0014 (15)	0.0028 (14)	0.0069 (15)
C22A	0.0210 (17)	0.0177 (19)	0.0288 (19)	0.0021 (15)	0.0042 (14)	0.0065 (15)
C22B	0.0197 (18)	0.020 (2)	0.033 (2)	0.0044 (15)	−0.0021 (14)	0.0090 (16)
C23A	0.0244 (19)	0.0125 (18)	0.0265 (18)	0.0000 (15)	−0.0014 (14)	0.0033 (15)
C23B	0.0218 (18)	0.0185 (19)	0.0298 (19)	0.0048 (15)	0.0058 (14)	0.0043 (15)
C24A	0.0214 (18)	0.0129 (18)	0.0252 (18)	0.0048 (14)	0.0019 (14)	0.0054 (14)
C24B	0.0193 (17)	0.0132 (18)	0.0234 (18)	−0.0029 (14)	0.0020 (13)	0.0053 (14)
C25A	0.0191 (17)	0.0175 (19)	0.0237 (18)	0.0078 (14)	0.0022 (13)	0.0039 (14)
C25B	0.0228 (18)	0.0172 (19)	0.0241 (18)	0.0020 (15)	0.0034 (14)	0.0069 (15)
O1A	0.0414 (15)	0.0224 (14)	0.0251 (13)	0.0043 (11)	0.0091 (11)	0.0046 (11)
O1B	0.0459 (15)	0.0258 (15)	0.0243 (13)	0.0077 (12)	0.0023 (11)	0.0046 (11)
O2A	0.0435 (15)	0.0201 (15)	0.0322 (15)	0.0027 (12)	0.0114 (11)	0.0006 (11)
O2B	0.0466 (15)	0.0243 (15)	0.0321 (14)	0.0122 (12)	−0.0023 (12)	−0.0007 (11)

### Geometric parameters (Å, °)

**Table:**

C1A—O1A	1.228 (4)	C12A—C13A	1.351 (4)
C1A—O2A	1.327 (4)	C12A—H12A	0.9300
C1A—C2A	1.454 (4)	C12B—C13B	1.340 (4)
C1B—O1B	1.218 (4)	C12B—H12B	0.9300
C1B—O2B	1.338 (4)	C13A—C14A	1.417 (4)
C1B—C2B	1.458 (4)	C13A—H13A	0.9300
C2A—C3A	1.345 (4)	C13B—C14B	1.427 (4)
C2A—H2A	0.9300	C13B—H13B	0.9300
C2B—C3B	1.347 (4)	C14A—C15A	1.390 (4)
C2B—H2B	0.9300	C14A—C25A	1.421 (4)
C3A—C4A	1.456 (4)	C14B—C15B	1.392 (4)
C3A—C9A	1.482 (5)	C14B—C25B	1.426 (4)
C3B—C4B	1.445 (4)	C15A—C16A	1.379 (4)
C3B—C9B	1.496 (4)	C15A—H15A	0.9300
C4A—C5A	1.340 (4)	C15B—C16B	1.375 (4)
C4A—H4A	0.9300	C15B—H15B	0.9300
C4B—C5B	1.352 (4)	C16A—C17A	1.387 (4)
C4B—H4B	0.9300	C16A—H16A	0.9300
C5A—C6A	1.441 (4)	C16B—C17B	1.385 (4)
C5A—H5A	0.9300	C16B—H16B	0.9300
C5B—C6B	1.435 (4)	C17A—C18A	1.395 (4)
C5B—H5B	0.9300	C17A—H17A	0.9300
C6A—C7A	1.344 (4)	C17B—C18B	1.395 (4)
C6A—H6A	0.9300	C17B—H17B	0.9300
C6B—C7B	1.348 (4)	C18A—C25A	1.421 (4)
C6B—H6B	0.9300	C18A—C19A	1.423 (4)
C7A—C10A	1.488 (4)	C18B—C25B	1.415 (4)
C7A—C8A	1.504 (4)	C18B—C19B	1.441 (4)
C7B—C10B	1.485 (4)	C19A—C20A	1.346 (4)
C7B—C8B	1.514 (4)	C19A—H19A	0.9300
C8A—H8A1	0.9600	C19B—C20B	1.335 (4)
C8A—H8A2	0.9600	C19B—H19B	0.9300
C8A—H8A3	0.9600	C20A—C21A	1.422 (4)
C8B—H8B1	0.9600	C20A—H20A	0.9300
C8B—H8B2	0.9600	C20B—C21B	1.435 (4)
C8B—H8B3	0.9600	C20B—H20B	0.9300
C9A—H9A1	0.9600	C21A—C22A	1.394 (4)
C9A—H9A2	0.9600	C21A—C24A	1.418 (4)
C9A—H9A3	0.9600	C21B—C22B	1.387 (4)
C9B—H9B1	0.9600	C21B—C24B	1.429 (4)
C9B—H9B2	0.9600	C22A—C23A	1.374 (4)
C9B—H9B3	0.9600	C22A—H22A	0.9300
C10A—C23A	1.384 (4)	C22B—C23B	1.370 (4)
C10A—C11A	1.424 (4)	C22B—H22B	0.9300
C10B—C23B	1.406 (4)	C23A—H23A	0.9300
C10B—C11B	1.419 (4)	C23B—H23B	0.9300
C11A—C24A	1.419 (4)	C24A—C25A	1.429 (4)
C11A—C12A	1.427 (4)	C24B—C25B	1.423 (4)
C11B—C24B	1.418 (4)	O2A—H2A1	0.876 (18)
C11B—C12B	1.442 (4)	O2B—H2B1	0.858 (18)
			
O1A—C1A—O2A	121.5 (3)	C13B—C12B—H12B	118.9
O1A—C1A—C2A	126.4 (3)	C11B—C12B—H12B	118.9
O2A—C1A—C2A	112.1 (3)	C12A—C13A—C14A	121.9 (3)
O1B—C1B—O2B	121.5 (3)	C12A—C13A—H13A	119.0
O1B—C1B—C2B	127.2 (3)	C14A—C13A—H13A	119.0
O2B—C1B—C2B	111.2 (3)	C12B—C13B—C14B	121.9 (3)
C3A—C2A—C1A	128.4 (3)	C12B—C13B—H13B	119.0
C3A—C2A—H2A	115.8	C14B—C13B—H13B	119.0
C1A—C2A—H2A	115.8	C15A—C14A—C13A	123.3 (3)
C3B—C2B—C1B	127.9 (3)	C15A—C14A—C25A	118.8 (3)
C3B—C2B—H2B	116.0	C13A—C14A—C25A	118.0 (3)
C1B—C2B—H2B	116.0	C15B—C14B—C25B	119.4 (3)
C2A—C3A—C4A	117.5 (3)	C15B—C14B—C13B	122.7 (3)
C2A—C3A—C9A	124.5 (3)	C25B—C14B—C13B	117.9 (3)
C4A—C3A—C9A	118.0 (3)	C16A—C15A—C14A	121.5 (3)
C2B—C3B—C4B	116.8 (3)	C16A—C15A—H15A	119.3
C2B—C3B—C9B	124.7 (3)	C14A—C15A—H15A	119.3
C4B—C3B—C9B	118.5 (3)	C16B—C15B—C14B	120.8 (3)
C5A—C4A—C3A	126.3 (3)	C16B—C15B—H15B	119.6
C5A—C4A—H4A	116.8	C14B—C15B—H15B	119.6
C3A—C4A—H4A	116.8	C15A—C16A—C17A	120.2 (3)
C5B—C4B—C3B	126.8 (3)	C15A—C16A—H16A	119.9
C5B—C4B—H4B	116.6	C17A—C16A—H16A	119.9
C3B—C4B—H4B	116.6	C15B—C16B—C17B	120.6 (3)
C4A—C5A—C6A	123.2 (3)	C15B—C16B—H16B	119.7
C4A—C5A—H5A	118.4	C17B—C16B—H16B	119.7
C6A—C5A—H5A	118.4	C16A—C17A—C18A	120.8 (3)
C4B—C5B—C6B	122.3 (3)	C16A—C17A—H17A	119.6
C4B—C5B—H5B	118.8	C18A—C17A—H17A	119.6
C6B—C5B—H5B	118.8	C16B—C17B—C18B	120.8 (3)
C7A—C6A—C5A	126.0 (3)	C16B—C17B—H17B	119.6
C7A—C6A—H6A	117.0	C18B—C17B—H17B	119.6
C5A—C6A—H6A	117.0	C17A—C18A—C25A	119.1 (3)
C7B—C6B—C5B	126.5 (3)	C17A—C18A—C19A	122.4 (3)
C7B—C6B—H6B	116.8	C25A—C18A—C19A	118.5 (3)
C5B—C6B—H6B	116.8	C17B—C18B—C25B	119.3 (3)
C6A—C7A—C10A	118.3 (3)	C17B—C18B—C19B	122.3 (3)
C6A—C7A—C8A	122.5 (3)	C25B—C18B—C19B	118.3 (3)
C10A—C7A—C8A	119.1 (3)	C20A—C19A—C18A	121.9 (3)
C6B—C7B—C10B	118.7 (3)	C20A—C19A—H19A	119.1
C6B—C7B—C8B	120.6 (3)	C18A—C19A—H19A	119.1
C10B—C7B—C8B	120.6 (3)	C20B—C19B—C18B	121.5 (3)
C7A—C8A—H8A1	109.5	C20B—C19B—H19B	119.3
C7A—C8A—H8A2	109.5	C18B—C19B—H19B	119.3
H8A1—C8A—H8A2	109.5	C19A—C20A—C21A	121.0 (3)
C7A—C8A—H8A3	109.5	C19A—C20A—H20A	119.5
H8A1—C8A—H8A3	109.5	C21A—C20A—H20A	119.5
H8A2—C8A—H8A3	109.5	C19B—C20B—C21B	121.4 (3)
C7B—C8B—H8B1	109.5	C19B—C20B—H20B	119.3
C7B—C8B—H8B2	109.5	C21B—C20B—H20B	119.3
H8B1—C8B—H8B2	109.5	C22A—C21A—C24A	118.6 (3)
C7B—C8B—H8B3	109.5	C22A—C21A—C20A	121.9 (3)
H8B1—C8B—H8B3	109.5	C24A—C21A—C20A	119.5 (3)
H8B2—C8B—H8B3	109.5	C22B—C21B—C24B	118.2 (3)
C3A—C9A—H9A1	109.5	C22B—C21B—C20B	122.5 (3)
C3A—C9A—H9A2	109.5	C24B—C21B—C20B	119.2 (3)
H9A1—C9A—H9A2	109.5	C23A—C22A—C21A	120.5 (3)
C3A—C9A—H9A3	109.5	C23A—C22A—H22A	119.7
H9A1—C9A—H9A3	109.5	C21A—C22A—H22A	119.7
H9A2—C9A—H9A3	109.5	C23B—C22B—C21B	121.3 (3)
C3B—C9B—H9B1	109.5	C23B—C22B—H22B	119.3
C3B—C9B—H9B2	109.5	C21B—C22B—H22B	119.3
H9B1—C9B—H9B2	109.5	C22A—C23A—C10A	122.5 (3)
C3B—C9B—H9B3	109.5	C22A—C23A—H23A	118.7
H9B1—C9B—H9B3	109.5	C10A—C23A—H23A	118.7
H9B2—C9B—H9B3	109.5	C22B—C23B—C10B	122.3 (3)
C23A—C10A—C11A	119.0 (3)	C22B—C23B—H23B	118.8
C23A—C10A—C7A	118.9 (3)	C10B—C23B—H23B	118.8
C11A—C10A—C7A	122.1 (3)	C21A—C24A—C11A	120.9 (3)
C23B—C10B—C11B	118.0 (3)	C21A—C24A—C25A	119.2 (3)
C23B—C10B—C7B	118.2 (3)	C11A—C24A—C25A	119.9 (3)
C11B—C10B—C7B	123.8 (3)	C11B—C24B—C25B	120.8 (3)
C24A—C11A—C10A	118.4 (3)	C11B—C24B—C21B	120.6 (3)
C24A—C11A—C12A	118.0 (3)	C25B—C24B—C21B	118.6 (3)
C10A—C11A—C12A	123.5 (3)	C14A—C25A—C18A	119.7 (3)
C24B—C11B—C10B	119.5 (3)	C14A—C25A—C24A	120.4 (3)
C24B—C11B—C12B	117.1 (3)	C18A—C25A—C24A	119.9 (3)
C10B—C11B—C12B	123.3 (3)	C18B—C25B—C24B	121.0 (3)
C13A—C12A—C11A	121.9 (3)	C18B—C25B—C14B	119.1 (3)
C13A—C12A—H12A	119.1	C24B—C25B—C14B	120.0 (3)
C11A—C12A—H12A	119.1	C1A—O2A—H2A1	113 (2)
C13B—C12B—C11B	122.2 (3)	C1B—O2B—H2B1	101 (3)
			
O1A—C1A—C2A—C3A	7.5 (5)	C25A—C18A—C19A—C20A	−3.1 (5)
O2A—C1A—C2A—C3A	−172.4 (3)	C17B—C18B—C19B—C20B	179.0 (3)
O1B—C1B—C2B—C3B	−7.0 (6)	C25B—C18B—C19B—C20B	0.7 (5)
O2B—C1B—C2B—C3B	173.2 (3)	C18A—C19A—C20A—C21A	1.0 (5)
C1A—C2A—C3A—C4A	177.5 (3)	C18B—C19B—C20B—C21B	−1.2 (5)
C1A—C2A—C3A—C9A	−2.7 (5)	C19A—C20A—C21A—C22A	−177.2 (3)
C1B—C2B—C3B—C4B	−178.5 (3)	C19A—C20A—C21A—C24A	1.7 (5)
C1B—C2B—C3B—C9B	0.1 (6)	C19B—C20B—C21B—C22B	−179.4 (3)
C2A—C3A—C4A—C5A	176.4 (3)	C19B—C20B—C21B—C24B	1.2 (5)
C9A—C3A—C4A—C5A	−3.4 (5)	C24A—C21A—C22A—C23A	−0.9 (4)
C2B—C3B—C4B—C5B	−176.9 (3)	C20A—C21A—C22A—C23A	178.0 (3)
C9B—C3B—C4B—C5B	4.4 (5)	C24B—C21B—C22B—C23B	−0.7 (5)
C3A—C4A—C5A—C6A	179.1 (3)	C20B—C21B—C22B—C23B	179.9 (3)
C3B—C4B—C5B—C6B	−178.8 (3)	C21A—C22A—C23A—C10A	2.2 (5)
C4A—C5A—C6A—C7A	177.3 (3)	C11A—C10A—C23A—C22A	−1.2 (5)
C4B—C5B—C6B—C7B	−177.5 (3)	C7A—C10A—C23A—C22A	179.8 (3)
C5A—C6A—C7A—C10A	−178.6 (3)	C21B—C22B—C23B—C10B	0.9 (5)
C5A—C6A—C7A—C8A	−2.3 (5)	C11B—C10B—C23B—C22B	−0.9 (5)
C5B—C6B—C7B—C10B	178.1 (3)	C7B—C10B—C23B—C22B	178.6 (3)
C5B—C6B—C7B—C8B	2.2 (5)	C22A—C21A—C24A—C11A	−1.3 (4)
C6A—C7A—C10A—C23A	51.1 (4)	C20A—C21A—C24A—C11A	179.8 (3)
C8A—C7A—C10A—C23A	−125.3 (3)	C22A—C21A—C24A—C25A	176.8 (3)
C6A—C7A—C10A—C11A	−127.8 (3)	C20A—C21A—C24A—C25A	−2.1 (4)
C8A—C7A—C10A—C11A	55.8 (4)	C10A—C11A—C24A—C21A	2.3 (4)
C6B—C7B—C10B—C23B	−43.4 (4)	C12A—C11A—C24A—C21A	178.1 (3)
C8B—C7B—C10B—C23B	132.5 (3)	C10A—C11A—C24A—C25A	−175.8 (3)
C6B—C7B—C10B—C11B	136.0 (3)	C12A—C11A—C24A—C25A	0.0 (4)
C8B—C7B—C10B—C11B	−48.1 (4)	C10B—C11B—C24B—C25B	−179.8 (3)
C23A—C10A—C11A—C24A	−1.0 (4)	C12B—C11B—C24B—C25B	3.6 (4)
C7A—C10A—C11A—C24A	177.9 (3)	C10B—C11B—C24B—C21B	−0.5 (5)
C23A—C10A—C11A—C12A	−176.6 (3)	C12B—C11B—C24B—C21B	−177.1 (3)
C7A—C10A—C11A—C12A	2.3 (5)	C22B—C21B—C24B—C11B	0.5 (5)
C23B—C10B—C11B—C24B	0.7 (5)	C20B—C21B—C24B—C11B	179.9 (3)
C7B—C10B—C11B—C24B	−178.8 (3)	C22B—C21B—C24B—C25B	179.8 (3)
C23B—C10B—C11B—C12B	177.0 (3)	C20B—C21B—C24B—C25B	−0.8 (5)
C7B—C10B—C11B—C12B	−2.4 (5)	C15A—C14A—C25A—C18A	−0.1 (4)
C24A—C11A—C12A—C13A	1.4 (4)	C13A—C14A—C25A—C18A	−178.4 (3)
C10A—C11A—C12A—C13A	177.0 (3)	C15A—C14A—C25A—C24A	179.1 (3)
C24B—C11B—C12B—C13B	−4.5 (5)	C13A—C14A—C25A—C24A	0.8 (4)
C10B—C11B—C12B—C13B	179.1 (3)	C17A—C18A—C25A—C14A	1.6 (4)
C11A—C12A—C13A—C14A	−1.8 (5)	C19A—C18A—C25A—C14A	−178.3 (3)
C11B—C12B—C13B—C14B	3.0 (5)	C17A—C18A—C25A—C24A	−177.6 (3)
C12A—C13A—C14A—C15A	−177.6 (3)	C19A—C18A—C25A—C24A	2.5 (4)
C12A—C13A—C14A—C25A	0.7 (4)	C21A—C24A—C25A—C14A	−179.2 (3)
C12B—C13B—C14B—C15B	179.8 (3)	C11A—C24A—C25A—C14A	−1.1 (4)
C12B—C13B—C14B—C25B	−0.7 (5)	C21A—C24A—C25A—C18A	0.0 (4)
C13A—C14A—C15A—C16A	176.8 (3)	C11A—C24A—C25A—C18A	178.1 (3)
C25A—C14A—C15A—C16A	−1.4 (5)	C17B—C18B—C25B—C24B	−178.7 (3)
C25B—C14B—C15B—C16B	0.3 (5)	C19B—C18B—C25B—C24B	−0.2 (5)
C13B—C14B—C15B—C16B	179.8 (3)	C17B—C18B—C25B—C14B	2.5 (5)
C14A—C15A—C16A—C17A	1.4 (5)	C19B—C18B—C25B—C14B	−179.1 (3)
C14B—C15B—C16B—C17B	0.2 (5)	C11B—C24B—C25B—C18B	179.6 (3)
C15A—C16A—C17A—C18A	0.2 (5)	C21B—C24B—C25B—C18B	0.3 (5)
C15B—C16B—C17B—C18B	0.6 (5)	C11B—C24B—C25B—C14B	−1.5 (5)
C16A—C17A—C18A—C25A	−1.7 (5)	C21B—C24B—C25B—C14B	179.2 (3)
C16A—C17A—C18A—C19A	178.2 (3)	C15B—C14B—C25B—C18B	−1.7 (5)
C16B—C17B—C18B—C25B	−2.0 (5)	C13B—C14B—C25B—C18B	178.8 (3)
C16B—C17B—C18B—C19B	179.7 (3)	C15B—C14B—C25B—C24B	179.5 (3)
C17A—C18A—C19A—C20A	177.0 (3)	C13B—C14B—C25B—C24B	−0.1 (5)

### Hydrogen-bond geometry (Å, °)

**Table:**

*D*—H···*A*	*D*—H	H···*A*	*D*···*A*	*D*—H···*A*
O2A—H2A1···O1A^i^	0.88 (2)	1.76 (2)	2.629 (3)	174 (4)
O2B—H2B1···O1B^ii^	0.86 (2)	1.79 (2)	2.624 (3)	162 (4)

Symmetry codes: (i) −*x*, −*y*+2, −*z*+1; (ii) −*x*+1, −*y*+3, −*z*+1.
